# Imaging of musculoskeletal injury: timing estimation and medico-legal issues

**DOI:** 10.1007/s11547-025-01990-4

**Published:** 2025-03-28

**Authors:** Giuseppe Davide Albano, Antonina Argo, Stefania Zerbo, Carlotta Scavone, Francesco Vitale, Carmelo Messina, Salvatore Gitto, Silvia Albano, Mauro Midiri, Paolo Vitali, Francesca Serpi, Giuseppe Lo Re, Massimo Galia, Cristina Cattaneo, Luca Maria Sconfienza, Domenico Albano

**Affiliations:** 1 Institute of Legal Medicine, Department of Health Promotion, Mother and Child Care, Internal Medicine and Medical Specialties, University of Palermo, Palermo, Italy; 2 U.O.C. Di Radiodiagnostica del P.O. S. Marta e S. Venera Acireale, ASP Catania, Catania, Italy; 3 Dipartimento di Scienze Biomediche per la Salute, Università degli Studi di Milano, Milan, Italy; 4 U.O.C. Radiodiagnostica, ASST Centro Specialistico Ortopedico Traumatologico Gaetano Pini-CTO, 20122 Milan, Italy; 5 IRCCS Istituto Ortopedico Galeazzi, 20161 Milan, Italy; 6 Department of Biomedicine, Neuroscience and Advanced Diagnostic (BI.N.D.), University of Palermo, 90127 Palermo, Italy; 7 Dipartimento di Scienze Biomediche, Chirurgiche ed Odontoiatriche, Università degli Studi di Milano, Milan, Italy

**Keywords:** Trauma, Injury, Forensic, Causality, Musculoskeletal imaging, Medico-legal issues

## Abstract

Musculoskeletal imaging plays a pivotal role in the evaluation of trauma, with applications spanning medical, forensic, and insurance contexts. Precisely dating musculoskeletal injuries is vital for reconstructing the timeline of events leading to trauma and verifying their accuracy. In forensic medicine, radiologists are frequently called upon by law enforcement and insurance companies to estimate the age of such injuries. This review aims to provide an overview of musculoskeletal imaging findings that can be used in medico-legal issues related to trauma to reach a comprehensive understanding of the causal relationship between the traumatic event and the clinical findings, with a particular focus on assessing causality, timing estimation and post-traumatic injury and impairment. Imaging plays a pivotal role in the precise and comprehensive evaluation of musculoskeletal traumatic injuries, with applications extending from immediate clinical care to legal and insurance considerations. Through various imaging modalities, it is possible to estimate the time elapsed since the injury and assess the impact of any pre-existing conditions. Effective collaboration between the forensic physician and the radiologist is essential to accurately determine the causal link between the injurious event and the resulting damage. This interdisciplinary approach ensures appropriate compensation and addresses the complex forensic aspects involved.

## Introduction

Musculoskeletal imaging plays a pivotal role in the evaluation of trauma, with applications spanning medical, forensic, and insurance contexts. Precisely dating musculoskeletal injuries is vital for reconstructing the timeline of events leading to trauma and verifying their accuracy [1]. In forensic medicine, radiologists are frequently called upon by law enforcement and insurance companies to estimate the age of such injuries. For living victims, the primary focus is on determining the time elapsed since the injury occurred [2, 3]. This information is critical in legal investigations, as it helps establish timelines and clarify the connection between the trauma and the resultant injury, particularly when discrepancies arise between medical evidence and the reported circumstances of the injury [4]. Current methods for injury dating rely on qualitative assessments, including clinical evaluations and imaging examinations. However, these methods can vary depending on the observer's expertise and the nature of the injuries. Forensic trauma analysis often depends on these assessments to support or refute claims regarding the timing of the injury [5]. Nonetheless, variability in the healing process—affected by factors such as the patient's age, pre-existing conditions, and the nature of the injury—poses challenges in standardizing the imaging-based estimation of musculoskeletal lesion age. Expert opinions, therefore, play a critical role in medico-legal cases, establishing the causal link between the alleged incident and the observed medical outcomes [6–8]. The objective of causal analysis in these contexts often involves both general and specific causality, which is a highly significant issue in forensic investigations [9]. This review aims to provide an overview of musculoskeletal imaging findings that can be used in medico-legal issues related to trauma to reach a comprehensive understanding of the causal relationship between the traumatic event and the clinical findings, with a particular focus on assessing causality, timing estimation and post-traumatic injury and impairment.

## Causal link with the accident and extent of damage

In contemporary practice, a cause is defined as an event or condition that is necessary for the effect to occur, adhering to the principle of temporal precedence. Causality, or the determination of cause-and-effect relationships, is integral to every legal case. As a result, the forensic physician is tasked with producing a forensic report that is both technically accurate and sufficiently comprehensive, applying widely accepted methodologies. However, these methods are not always evidence-based or standardized [10–12]. Imaging examinations remain a fundamental tool in determining the causal link between an incident, such as a motor vehicle accident, and the injuries sustained [13, 14]. Indeed, imaging can reveal:

- The nature of the injuries: Certain injuries are characteristic of high-energy trauma, while others may suggest minor impact trauma.
- The distribution of injuries: The anatomical location of injuries may correspond to the dynamics of the accident (e.g., injuries typical of seatbelt or airbag impact).
- Compatibility with the accident dynamics: Imaging can verify whether the injuries align with the type of accident described.

Furthermore, imaging techniques facilitate a thorough assessment of injury severity, fulfilling several critical functions:

- Damage assessment: Precisely evaluate the severity of injuries and their impact on the individual's health and functional capacity.
- Prognosis: Provide forecasts regarding recovery duration and potential long-term complications.
- Evaluation of biological damage: Identify permanent impairments, crucial for calculating compensation in medico-legal and insurance settings.
- Monitoring recovery: Track the healing process and assess the effectiveness of treatments over time, ensuring timely and appropriate clinical interventions.

One of the most widely accepted methods for assessing injury severity is the Abbreviated Injury Scale (AIS), an internationally recognized system that qualitatively categorizes the extent of individual injuries [15–17]. The AIS assigns a score ranging from 1 to 9, reflecting the "threat to life" associated with a specific injury in different body regions. Scores range from minor injuries, such as sprains and bruises, to fatal trauma. Additionally, the Injury Severity Score (ISS) evaluates the overall trauma severity by assigning a cumulative score from 0 (no injury) to 75 (fatal injury) [18, 19]. These scales are invaluable for trauma assessment and treatment planning. After determining the type of injury, it is essential to assess whether the damage is compensable and permanent:

- Transient damage: Refers to temporary injuries that resolve within a relatively short period after the causative event.
- Permanent damage: Involves injuries with lasting consequences, where complete restoration of psychophysical integrity is unattainable, with either partial or total impairment.

These categories are encompassed within the concept of non-pecuniary damage, which refers to harm that affects non-economic aspects, such as biological or moral injury, and is compensated through financial restitution [20, 21]. To properly assess such injuries and support legal claims, it is necessary to provide evidence of legally recognized harm and the resultant losses. The claimant must supply documentation outlining the circumstances of the injury, the consequent damage, and the criteria used for compensation. The burden of proof may be met through various forms of evidence, including documents, witness testimonies, and expert technical evaluations.

## Imaging

Imaging techniques such as X-rays, CT, and MRI play a crucial role in determining the timing of musculoskeletal injuries by revealing the stages of the healing process. Ultrasound, on the other hand, is barely used in medico-legal contexts, primarily because it does not provide an objective record of images that can be easily reviewed by another expert for a second opinion. Radiologists are increasingly consulted as expert evaluators by private individuals, courts, and insurance companies to assess musculoskeletal injuries resulting from trauma. Their role is to establish the causal relationship between the traumatic event and the injury, considering both the mechanics and energy of the trauma, as well as its temporal correlation to the incident. In addition to establishing causality, radiologists may also be required to assess the presence of pre-existing pathological conditions that could have partially or entirely contributed to the injury (e.g., degenerative rotator cuff tendinopathy as a contributing factor in a supraspinatus tendon tear). Identifying these pre-existing conditions is essential, as they may influence compensation eligibility. Consequently, radiologists must possess comprehensive knowledge of the clinical features and temporal evolution of both direct and indirect signs of traumatic injuries to bones, muscles, tendons, fibrocartilage, and capsuloligamentous structures to provide an accurate assessment of damage. Common areas frequently addressed in radiological consultations within the insurance and medico-legal fields include fractures and injuries to the spine, shoulder, knee, and ankle.

### Fracture

Radiography is commonly employed as the first-level imaging modality in emergency settings, with CT serving as a secondary diagnostic tool in ambiguous cases or for evaluating complex anatomical regions such as the scapula, midfoot, pelvis, and spine [22]. MRI is not typically used during the initial emergency assessment unless specific clinical circumstances, such as neurologic emergencies involving spinal cord compression due to vertebral fractures, are present. However, MRI can be especially valuable in young patients for detecting subtle fractures that may be missed on radiographs. In such cases, non-displaced fractures of the scaphoid, ribs, midfoot, and elbow are often overlooked on initial radiographs and identified later with MRI and/or CT [23].

**Fig. 1:**
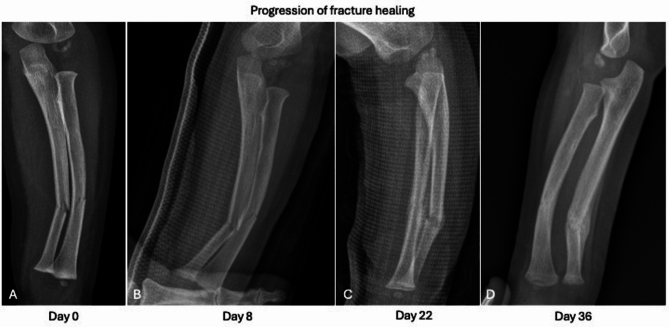
Progression fracture healing. Fracture of ulnar and radial shaft from day 0 (**A**), to day 8 (**B**), day 22 (**C**), and day 36 (**D**)

Radiographic indicators of acute fractures include cortical and trabecular bone disruption, fragment displacement, soft tissue swelling, changes in periarticular fat pads, and sclerotic lines resulting from trabecular thickening. CT offers a more detailed and precise assessment of these findings. A fracture presenting with sharp lines and no evidence of callus formation or bone bridging can be classified as acute, as bone healing processes have not yet commenced [24]. Periosteal reaction, often the earliest sign of bone healing, appears after approximately 2 weeks in younger individuals [25], occurring even earlier in children under 5 years of age [26], when sclerosis of bone margins is not yet detectable. During the first 2 weeks post-injury, radiographs may show increased lucency of the fracture line, giving the appearance of pseudo-widening. As healing progresses, the fracture margins become sclerotic, bone bridging occurs, and gaps are filled [27, 28]. In adults, complete radiographic healing typically takes between 4 to 12 weeks depending on the affected bone (i.e., 3–4 weeks for phalanges, 10–12 weeks for femoral neck fractures), though healing occurs more rapidly in younger patients (Fig. 1) [29, 30].

MRI adds significant value by identifying bone edema and providing detailed evaluation of soft tissues, including muscles, tendons, ligaments, capsules, fibrocartilage, and cartilage [31, 32]. While bone edema is traditionally associated with recent fractures, this finding can persist for several months, or even up to a year post-trauma, even after the fracture has fully healed and is no longer visible on radiographs or CT scans [33]. Furthermore, it is important to note that its extent may increase during the initial days or weeks as a result of increased vascularization and healing processes.

**Fig. 2:**
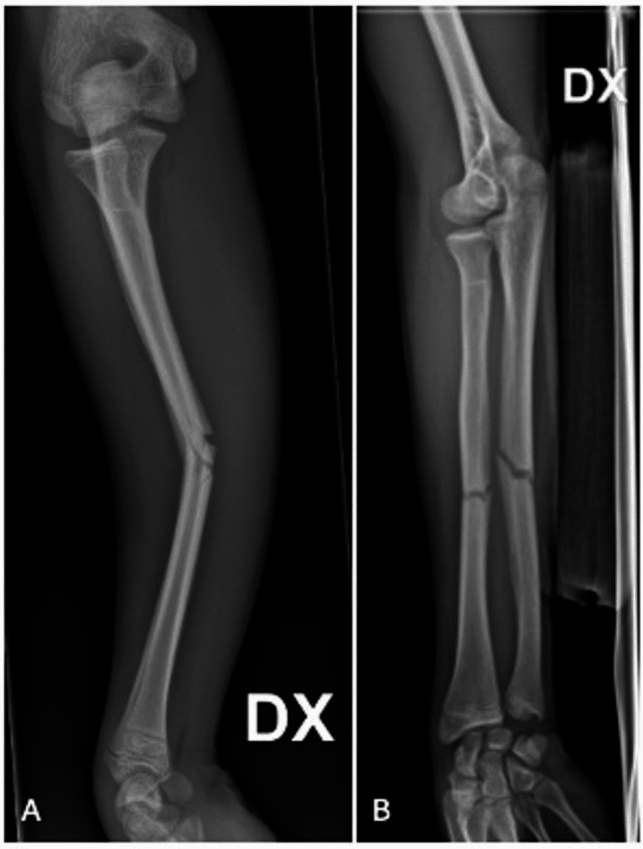
Self-inflicted fracture. Forearm transverse displaced fracture of a 17-year-old male patient induced by his father for the purpose of insurance fraud. Initially, the fracture was attributed to a car accident

A topic warranting separate consideration is that of self-inflicted bone fractures. These injuries are typically self-induced or orchestrated by criminal organizations for the purpose of insurance fraud. It is essential that radiologists recognize the atypical fracture patterns and discrepancies between the nature and severity of the injuries and the patient’s reported history, which is often attributed to a traffic accident [34]. These fractures mostly involve the shaft of long bones, particularly of humerus, radius, ulna, tibia, and fibula. The typical pattern is a transverse fracture of the diaphysis, often displaced, with possible comminution or small third fragment due to a compressive force applied (Fig. 2).

### Spine

**Fig. 3:**
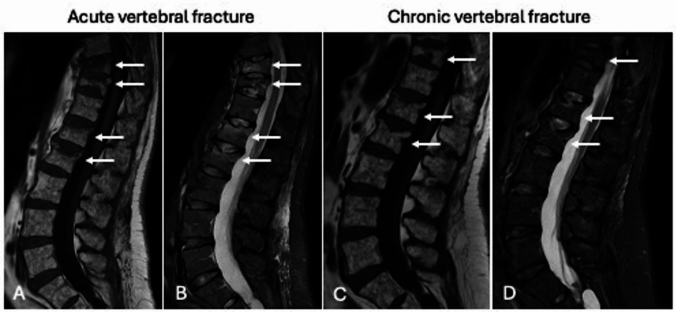
Vertebral fractures. MRI performed five days after trauma (**A**, **B**) show acute vertebral fractures (arrows) of T10, T11, L1, and L2, with bone edema appearing as bands along the collapsed endplates on STIR image (**B**) and associated with hypointense striae indicative of trabecular thickening on T1-weighted image (**A**). Spine MRI performed six months after trauma (T1-weighted on **C**, STIR image on **D**) shows chronic fractures (arrows) of T10, T11, L1, and L2, without bone edema

Special attention must be given to the timing of vertebral fractures due to the unique diagnostic challenges and specific findings associated with this anatomical region. MRI is frequently employed not only to detect bone edema at the fracture site [35] but also because it is the most effective imaging modality for assessing intervertebral discs and ligaments [36]. In severe acute fractures, paravertebral hematomas (either endo- or extra-canal) and ligament injuries may also be evident [37]. However, bone edema is not highly specific for identifying acute or recent fractures, as it can persist for several months after the fracture occurs, as abovementioned [35]. Hence, the absence of bone edema can be a valuable indicator for distinguishing chronic fractures from more recent ones. Typically, edema appears as a band along the collapsed endplate and is often associated with hypointense striae indicative of trabecular thickening, except in cases of complex fractures where diffuse vertebral edema is present [38]. In this context, it is essential to differentiate post-traumatic bone edema from degenerative disc-related edema (Modic Type 1). This distinction can be made by carefully evaluating the disc and endplate, bearing in mind that bone edema extending to the vertebral pedicles is characteristic of fractures and is not observed in Modic Type 1 changes. Another key feature seen in follow-up imaging (including X-rays, CT, and MRI) of recent, non-healed fractures is the progression of vertebral collapse along with the development of sclerotic changes in the fractures site. In contrast, chronic fractures may be accompanied by degenerative changes, such as osteophytes, disc thinning, and dehydration, which can appear in two or multiple spinal segments near the fracture site. These changes result from chronic biomechanical stress induced by the fracture-related vertebral deformity (Fig. 3).

**Fig. 4:**
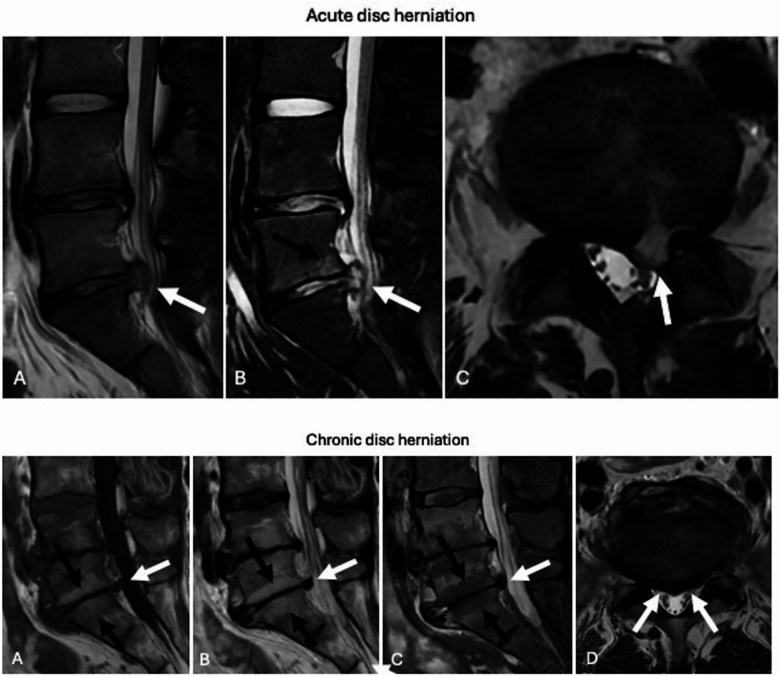
Acute L5-S1 disc herniation (white arrows) with high signal on sagittal T2-weighted (**A**), sagittal STIR (**B**), and axial T2-weighted (**C**) images, due to the high degree of hydration. No reactive osteophyte is observed, while it is possible to see bone edema of L5 close to the herniated disc on sagittal STIR image (**B**, black arrow). Chronic L5-S1 disc herniation (white arrows) with low signal on sagittal T1-weighted (**A**), sagittal T2-weighted (**B**), sagittal STIR (**C**), and axial T2-weighted (**D**) images, due to the low degree of hydration. Reactive osteophyte is not associated with bone edema. Modic 2 fatty changes (black arrows) of the bone adjacent to the endplates is observed with high signal on T1-weighted (**A**) and T2-weighted (**B**) images, and low signal on STIR (**C**)

The assessment of the timing of disc herniations presents even greater complexity, and in some cases, it is not possible to provide a definitive judgment. Nonetheless, the following general observations can be made. Acute herniations typically exhibit a higher degree of hydration compared to chronic herniations, which is reflected by a high signal intensity of the herniated disc material on T2-weighted and STIR sequences. Moreover, a rupture of the annulus fibrosus, accompanied by edematous changes in the nucleus pulposus can be observed. Furthermore, acute herniations may coexist with other acute traumatic injuries, such as fractures and ligament damage, aiding in the estimation of the timing. Conversely, chronic disc herniations are often associated with reactive osteophyte formation, which develops around the herniated disc (Fig. 4).

### Shoulder

**Fig. 5:**
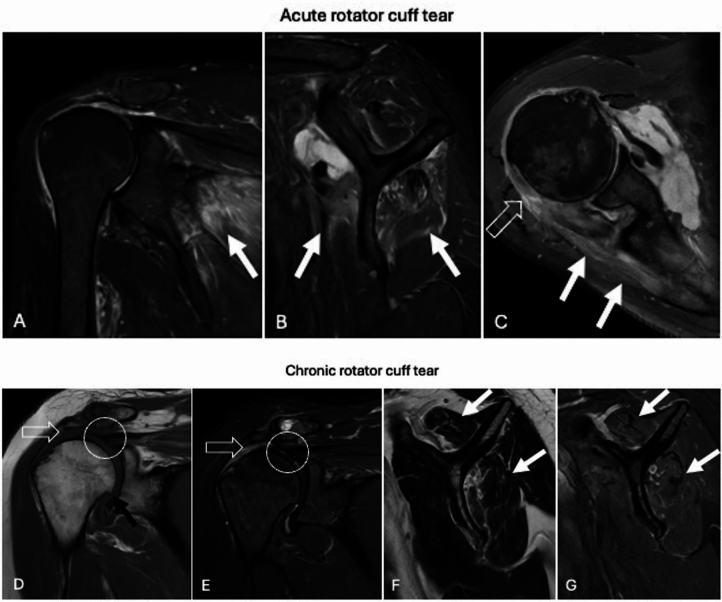
Acute rotator cuff tear. Shoulder MRI performed 6 days after trauma with fat-suppressed proton density weighted images (**A**, **B**, **C**) show edematous and frayed retracted infraspinatus tendon (**C**, void arrow), intra- and peri-muscular edema (**A**, **B**, **C**; arrows). Muscle trophism is well-preserved and the glenohumeral joint does not show degenerative changes. Chronic rotator cuff tear. Shoulder MRI performed eleven months after trauma show complete supraspinatus tendon tear with massive retraction of the tendon that appear rounded, atrophic, and hypointense (circle) on coronal T1-weighted (**D**) and fat-suppressed proton density weighted image (**E**). Mild atrophy and fat infiltration (white arrows) without inter- and/or peri-muscular edema can be noted in supraspinatus and infraspinatus muscles on sagittal T2-weighted (**F**) and fat-suppressed proton density weighted (**G**) images. Glenohumeral osteoarthritis (**D**; black arrow), humeral cranialization and reduction of the subacromial space (void arrows) are observed

Discriminating between traumatic and degenerative causes of rotator cuff injuries presents significant challenges, as tendon damage typically occurs against a backdrop of pre-existing degeneration. In such cases, trauma often acts as a contributing factor. Notably, approximately 25% of individuals over the age of 60 and 50% of those over 70 exhibit rotator cuff tendon injuries [39]. In estimating the timing of rotator cuff tears, several findings suggest that a rupture is acute [40, 41]. Generally, retracted tendons appear edematous and frayed, mild tendon retraction is observed, associated intra- and/or peri-muscular edema is present, muscle trophism is well-preserved, the humeral head remains in its normal position, and the glenohumeral joint shows no signs of degenerative changes, unless pre-existing conditions such as instability or inflammatory disorders are present (Fig. 5) [40]. Conversely, chronic cuff tears typically present the following characteristics: retracted tendons appear rounded, atrophic, and hypointense, no inter- and/or peri-muscular edema is noted, decreased cuff muscles trophism, humeral cranialization, obliteration of the subacromial space, acromial remodeling, and glenohumeral osteoarthritis can be seen, particularly in cases of massive chronic cuff tears (Fig. 5) [42, 43].

Notably, the presence of effusion in the glenohumeral joint or subacromial–subdeltoid bursa is not a reliable indicator for determining the timing of a traumatic rotator cuff injury, as these features may occur during either the acute or chronic phases and are often associated with degenerative or inflammatory conditions. However, the absence of effusion in both the joint and bursa decreases the likelihood of an acute tendon rupture. Additionally, depending on the trauma dynamics (e.g., blunt, distracting, with or without dislocation), post-traumatic bone edema may be observed in the acute–subacute phase, such as edema on the posterior–superior aspect of the humeral head in Hill–Sachs lesions resulting from anterior glenohumeral dislocation. Of note, also chronic rotator cuff tears can cause although indirectly bone marrow edema (i.e., with high pressure subchondral cysts), so the distribution of bone edema and any coexisting cyst should be considered to assess the reason of edema itself.

### Knee

The estimation of the timing of capsuloligamentous and meniscal knee injuries relies on both direct signs (appearance of the affected structure) and indirect signs (appearance of bone, cartilage, periarticular soft tissues, and effusion). While MRI can provide reliable information on the timing of capsuloligamentous injuries, the same level of certainty cannot be applied to many meniscal tears.

**Fig. 6:**
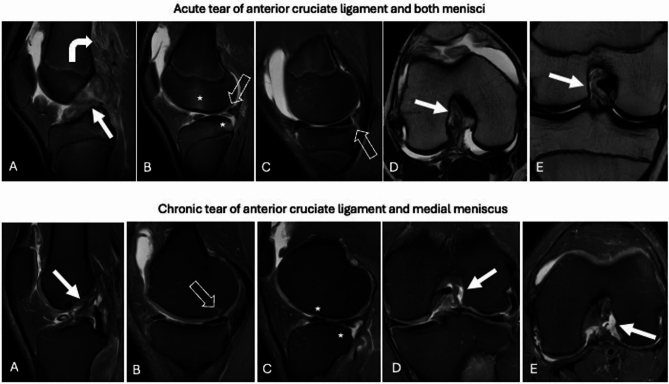
Acute tear of anterior cruciate ligament and both menisci. Knee MRI performed 2 days after trauma shows edematous and thickened appearance of the anterior cruciate ligament with focal irregularity of the bundles (white arrows) on sagittal fat-suppressed proton density weighted (**A**), axial (**D**) and coronal (**E**) T2-weighted images. Indirect signs of acute tear are peri-capsular edema (**A**; curved arrow) and lateral femoro-tibial bone edema (B; asterisks). Also note the radial tear of the lateral meniscus (**B**; void arrow) and meniscocapsular separation (**C**; void arrow). Chronic tear of anterior cruciate ligament and medial meniscus. Knee MRI performed 5 months after trauma shows proximal avulsion and thinning of the anterior cruciate ligament (white arrows) on sagittal (**A**), coronal (**D**), and axial (**E**) fat-suppressed proton density weighted images, without bone edema (**C**; asterisks). Also note chondral injury of the medial femoral condyle (**B**; void arrow) and medial meniscus tear (**B**)

**Fig. 7:**
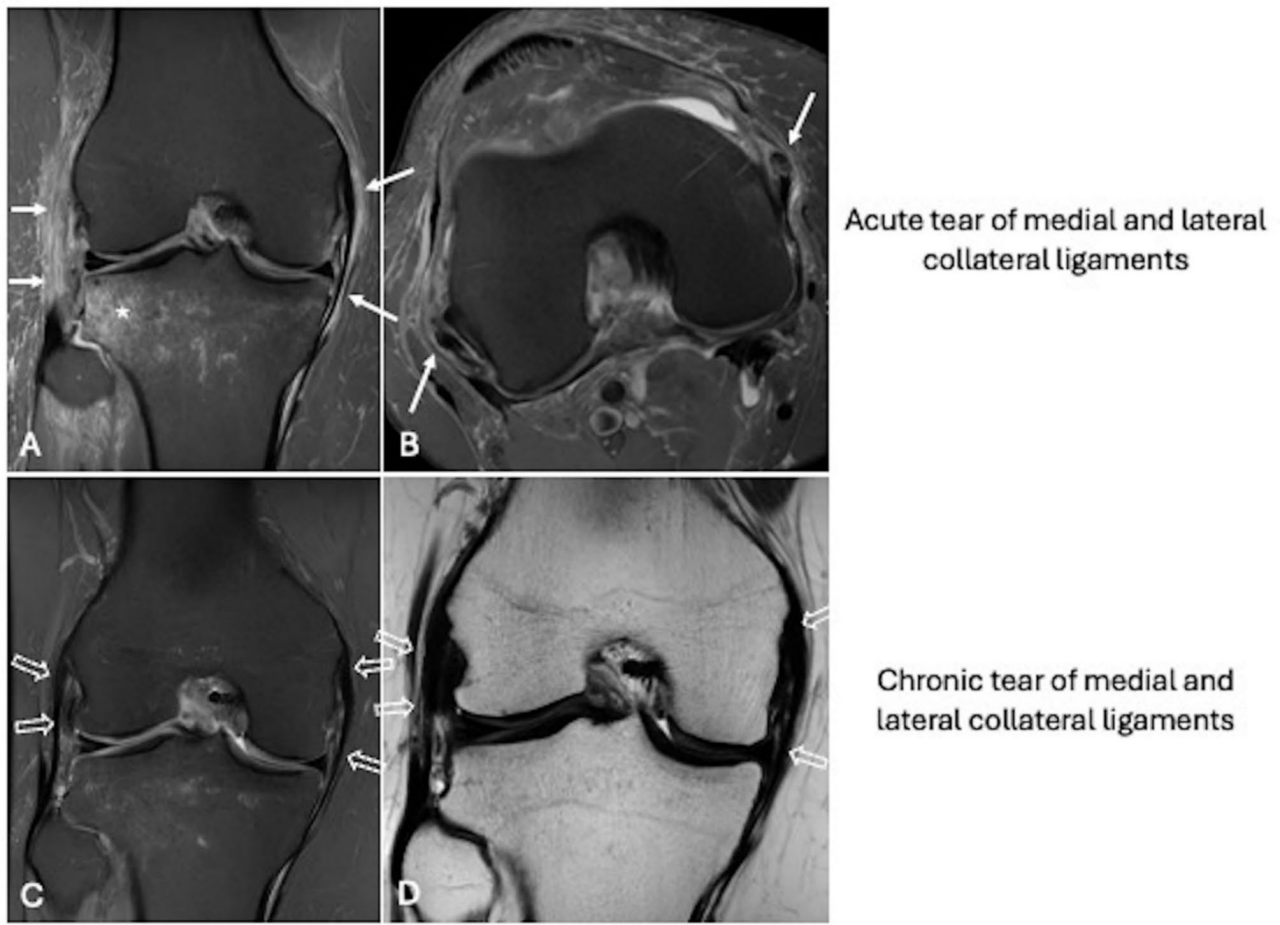
Acute and chronic tear of collateral ligaments. Knee MRI performed 12 days after trauma shows acute tear of medial and lateral collateral ligaments presenting with diffuse peri-ligamentous edema (white arrows) on coronal (**A**) and axial (**B**) fat-suppressed proton density weighted images, peri-capsular edema, and lateral tibia bone edema (**A**; asterisk). Knee MRI performed 5 months after trauma shows chronic tear of medial and lateral collateral ligaments presenting with hypointense thickening of both ligaments (**C**, **D**; void arrows) with neither peri-ligamentous edema, nor peri-capsular edema, nor bone edema

In acute capsuloligamentous injuries, such as those affecting the cruciate and collateral ligaments, the following findings are typically observed [44] (Figs. 6, 7): (i) edematous and thickened appearance of the ligament bundles and capsule; (ii) avulsion, focal disruption, and/or irregularity of the ligament bundles; (iii) peri-ligamentous and peri-capsular edema; (iv) bone edema due to contusion or traction in characteristic locations (e.g., lateral femoro-tibial bone in pivot shift trauma, medial epicondyle at the insertion of the collateral ligament in valgus stress, anterior tibia in posterior cruciate ligament injuries), sometimes accompanied by osteochondral or subchondral fractures. In contrast, MRI findings in chronic capsuloligamentous injuries include [45] (Figs. 6, 7): (i) thinning of the anterior cruciate ligament in partial tears and atrophy of the ligament bundles in complete tears; (ii) progressive bowing of the posterior cruciate ligament and potential anterior tibial translation due to anterior cruciate ligament insufficiency; (iii) hypointense scar thickening of the posterior cruciate ligament in partial tears or focal reductions in thickness, also hypointense; (iv) hypointense thickening of the collateral ligaments in all sequences; (v) absence of peri-ligamentous and peri-capsular edema; (vi) absence of bone edema; (vii) detachment of bone fragments or post-traumatic bone deformities without associated edema; (viii) ossification at proximal insertion of the medial collateral ligament (Pellegrini–Stieda lesion).

**Fig. 8:**
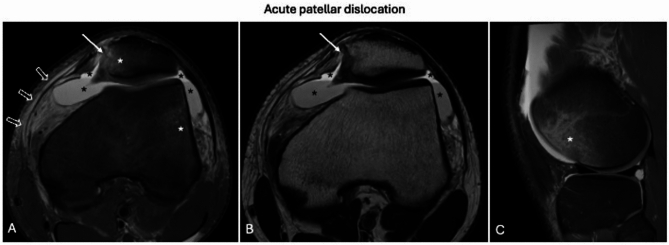
Acute patellar dislocation. Knee MRI performed 4 days after trauma shows bone edema in the inferomedial aspect of the patella (**A**; white asterisk) and the anterolateral aspect of the lateral condyle (**C**; white asterisk), with osteochondral fracture of the patella (**A**, **B**; white arrows). Also note the disruption of the medial patellofemoral ligament with peri-ligamentous edema (**A**; void arrows) and joint effusion with hemarthrosis (**B**, **C**; black asterisks)

In cases of acute patellar dislocation, bone edema is commonly observed on the inferomedial aspect of the patella and the anterolateral aspect of the lateral condyle, findings that may persist for several months following the dislocation. These findings may be accompanied by chondral lesions, fractures, or osteochondral detachments. Additional acute-phase findings include edema, thickening, and disruption of the medial patellofemoral ligament, edema at its epicondylar insertion, and joint effusion with hemarthrosis in cases of associated fractures [33] (Fig. 8).

Unfortunately, imaging offers limited assistance in estimating the timing of meniscal tears. Nonetheless, it is possible to estimate timing in certain types of meniscal tears, particularly those frequently associated with other capsuloligamentous injuries, such as peripheral vertical tears of the posterior horn of both menisci or bucket handle injuries, which are often seen in conjunction with anterior cruciate ligament tears [46] (Fig. 6). In differentiating between traumatic and degenerative meniscal tears, some factors may suggest a traumatic origin, such as younger age, the absence of degenerative changes in the meniscus (evidenced by a lack of diffuse hyperintensity, irregularity, fibrillation, or fragmentation), and the absence of degenerative changes in the ipsilateral femoro-tibial compartment, as seen in advanced osteoarthritis. As with rotator cuff injuries, the presence of joint effusion is not a specific indicator of an acute injury to the cruciate ligament or menisci, as it can be present in both acute and chronic phases. However, the absence of joint effusion reduces the likelihood of an acute injury.

### Ankle

**Fig. 9:**
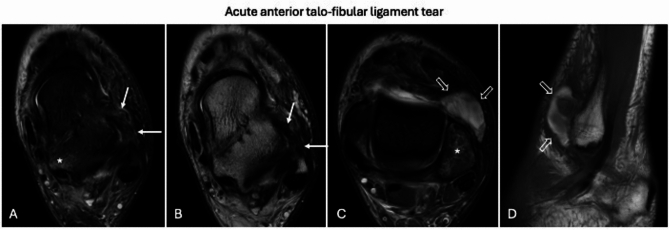
Acute anterior talo-fibular ligament partial tear. Ankle MRI performed 18 days after trauma shows edematous and thickened appearance of anterior talo-fibular ligament (**A**, **B**; white arrows) with peri-capsular edema, talus (**A**) and lateral malleolus (**C**) bone edema (asterisks). Also note the anterolateral hematoma (void arrows) with high signal on axial fat-suppressed proton density weighted (**C**) and sagittal T1-weighted (**D**) images

**Fig. 10:**
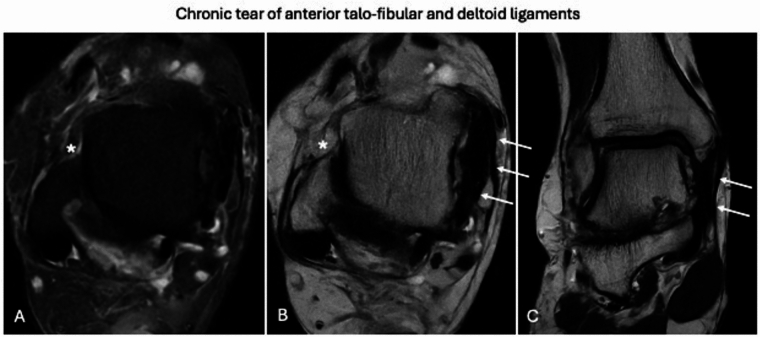
Chronic tear of anterior talo-fibular and deltoid ligaments. Ankle MRI performed four months after trauma shows anterior talo-fibular ligament atrophy, which is unidentifiable on axial fat-suppressed proton density weighted (**A**) and T2-weighted (**B**) images. The hypointense fibroscar thickening of deltoid ligament can be recognized on axial T2-weighted (**B**) and coronal T2-weighted (**C**) images. Neither bone edema nor peri-capsular edema can be seen on axial fat-suppressed proton density weighted (**A**) image

In the context of estimating the timing of ligament injuries in the tibiotalar joint and tibiofibular syndesmosis, many of the considerations applicable to knee ligaments are similarly relevant [47]. The following imaging findings are typically associated with acute ligament tears [47] (Fig. 9): (i) an edematous and thickened appearance of the ligaments; (ii) peri-ligamentous and peri-capsular edema; (iii) avulsion, focal disruption, and irregularity of the ligament bundles; and (iv) bone edema, especially when accompanied by osteochondral or subchondral fractures. In contrast, the recognized imaging features of chronic capsuloligamentous injuries include [48] (Fig. 10): (i) hypointense fibroscar thickening of the ligaments across all sequences; (ii) ligament atrophy, often with unidentifiable ligament stumps (particularly common in lateral ligaments); (iii) absence of peri-ligamentous and peri-capsular edema; (iv) absence of bone edema; and (v) the presence of bone fragments or post-traumatic bone deformities without associated edema. Additionally, in the ankle, the absence of joint effusion further increases the likelihood of chronic capsuloligamentous injuries. Notably, in deltoid ligament injuries, the loss of the normal striated appearance is related to the replacement of fat between bundles by fibrotic tissue (hypointense on all pulse sequences) in chronic tears and by edema (hyperintense on fluid sensitive sequences) in acute tears.

## Conclusions

Imaging plays a pivotal role in the precise and comprehensive evaluation of musculoskeletal traumatic injuries, with applications extending from immediate clinical care to legal and insurance considerations. Through various imaging modalities, it is possible to estimate the time elapsed since the injury and assess the impact of any pre-existing conditions. Effective collaboration between the forensic physician and the radiologist is essential to accurately determine the causal link between the injurious event and the resulting damage. This interdisciplinary approach ensures appropriate compensation and addresses the complex forensic aspects involved.

## Data Availability

Not applicable. No new data have been created.
